# Moderate Exercise Training Attenuates the Severity of Experimental Rodent Colitis: The Importance of Crosstalk between Adipose Tissue and Skeletal Muscles

**DOI:** 10.1155/2015/605071

**Published:** 2015-01-05

**Authors:** Jan Bilski, Agnieszka I. Mazur-Bialy, Bartosz Brzozowski, Marcin Magierowski, Katarzyna Jasnos, Gracjana Krzysiek-Maczka, Katarzyna Urbanczyk, Agata Ptak-Belowska, Malgorzata Zwolinska-Wcislo, Tomasz Mach, Tomasz Brzozowski

**Affiliations:** ^1^Department of Physical Exercise, Faculty of Health Care, Jagiellonian University Medical College, 20 Grzegorzecka Street, 31-531 Cracow, Poland; ^2^Gastroenterology and Hepatology Clinic, Jagiellonian University Medical College, 5 Sniadeckich Street, 31-501 Cracow, Poland; ^3^Department of Physiology, Faculty of Medicine, Jagiellonian University Medical College, 16 Grzegorzecka Street, 31-531 Cracow, Poland; ^4^Department of Pathomorphology, Faculty of Medicine, Jagiellonian University Medical College, 16 Grzegorzecka Street, 31-531 Cracow, Poland

## Abstract

Although progress has been recently made in understanding of inflammatory bowel diseases (IBD), their etiology is unknown apart from several factors from adipose tissue and skeletal muscles such as cytokines, adipokines, and myokines were implicated in the pathogenesis of ulcerative colitis. We studied the effect high-fat diet (HFD; cholesterol up to 70%), low-fat diet (LFD; cholesterol up to 10%), and the normal diet (total fat up to 5%) in rats with TNBS colitis forced to treadmill running exercise (5 days/week) for 6 weeks. In nonexercising HFD rats, the area of colonic damage, colonic tissue weight, the plasma IL-1*β*, TNF-*α*, TWEAK, and leptin levels, and the expression of IL-1*β*-, TNF-*α*-, and Hif1*α* mRNAs were significantly increased and a significant fall in plasma adiponectin and irisin levels was observed as compared to LFD rats. In HFD animals, the exercise significantly accelerated the healing of colitis, raised the plasma levels of IL-6 and irisin, downregulated the expression of IL-1*β*, TNF-*α*, and Hif1*α*, and significantly decreased the plasma IL-1*β*, TNF *α*, TWEAK, and leptin levels. We conclude that HFD delays the healing of colitis in trained rats *via* decrease in CBF and plasma IL-1*β*, TNF-*α*, TWEAK, and leptin levels and the release of protective irisin.

## 1. Introduction 

Inflammatory bowel diseases (IBD) such as ulcerative colitis (UC) and Crohn's disease (CD) are characterized by chronic relapsing inflammation of the gastrointestinal (GI) tract. Their etiology still remains unknown and it is believed that a combination of environmental agents and a dysfunctional mucosal immune system in genetically susceptible individuals could play an important role in their development [1, 2]. The composition of the gut microbiome could be an important environmental factor in IBD and a number of studies suggested a mucosal immune response to commensal bacteria in the pathogenesis of IBD and particularly in CD. The typical inflammatory response starts with an infiltration of neutrophils and macrophages known to release a variety of cytokines and chemokines that aggravate the immune response. It was established that CD is a Th1 cytokine-mediated disease characterized by increased production of interferon- (IFN-) *γ*, while UC bears a resemblance to a modified Th2 profile, with an augmented release of interleukin- (IL-) 5 but normal IFN-*γ* production [3]. Cytokines, such as TNF-*α*, IL-1*β*, and IL-6, that are more promiscuous in their function, are associated with both forms of IBD to a lesser or greater degree [3, 4]. Each of these cytokines activates NF-*κ*B and the mitogen-activated protein (MAP) kinases and induces various “downstream” proinflammatory effects responsible for the tissue and organ pathology in IBD [3]. Epidemiological studies have indicated that the incidence and prevalence of IBD rapidly increased particularly in Western countries and the rise observed in the rest of the world closely correlates with adopting a Western lifestyle including dietary habits, physical inactivity, and obesity [5]. Although the majority of CD patients are undernourished, there is now evidence that increasing body mass index (BMI) and overweight are emerging features of CD and may be associated with more severe course of disease activity [6, 7]. In Crohn's disease-relevant mouse model, the high-fat diet feeding actually accelerated the intestinal inflammation [8]. Characteristic feature for CD is hypertrophy of the mesenteric fat tissue located around the inflamed parts of the intestine [9]. Recent research suggests that this fat wrapping contributes actively to disease severity and may influence onset of complications [10–17]. This mesenteric fat is present from the onset of disease and is associated with overexpression of TNF-*α*, leptin, and other adipokines and correlates with the severity of intestinal inflammation and tissue injury, suggesting an important role for adipose tissue in the intestinal inflammatory process in CD [9]. Well-documented observations that physical activity is correlated inversely with systemic low-level inflammation lead to the suggestion that the anti-inflammatory activity induced by regular exercise may be responsible for some beneficial health effects in patients with chronic diseases including IBD [18]. Exercise may exert its anti-inflammatory effect* via* a reduction in visceral fat mass and/or by induction of an anti-inflammatory environment with each bout of exercise. Such effects may in part be mediated* via* muscle-derived peptides, so-called “myokines” [19].

While no single animal model fully captures the clinical and histopathological features of human IBD, the 2,4,6-trinitrobenzenesulfonic acid- (TNBS-) induced colitis is believed to be the most relevant to study CD-related immune responses [20]. The intrarectal administration of TNBS hapten reagent in ethanol solution causes disruption of the epithelial layer and exposes the* lamina propria* to bacterial and host haptenized protein. The experimental colitis induced by TNBS has many of the typical features of CD including the severe transmural inflammation, diarrhea, weight loss, and induction of IL-12-driven inflammation with a massive Th-1-mediated response [20]. In rodents with TNBS-induced colitis, a characteristic reduction of food intake and loss of weight together with a decrease in skeletal muscle mass associated with the severity of colitis have been described [21].

Our present study was designed to determine the effect of moderate forced treadmill on experimental colitis caused by intrarectal administration of TNBS in rats fed normal, low-fat, or high-fat diet. We hypothesized that diet-induced obesity augments the severity of experimental colitis and that the possible beneficial effects of physical exercise contribute to healing of colitis by modifying muscle-adipose tissue crosstalk. This particular issue has not been so far closely elucidated in relation to different diets and the possible alterations in plasma levels of myokines and adipokines and the expression of proinflammatory factors (cytokines and hypoxia inducible factor-1 alpha) considered as markers of inflammatory reactions associated with development and healing of colitis.

## 2. Material and Methods

### 2.1. Studies in Animal Model

Animal studies were carried out on eighty male Wistar rats 200–220 g with free access to water and food and adapted to laboratory conditions and 12/12 h day/night cycles. The study was approved by the local Ethical Committee at the Jagiellonian University Medical College in Cracow, Poland, and run in accordance with the Helsinki declaration.

One hundred twenty animals were randomized into the three experimental series (A–C) and fed for 8 weeks with either (A) high-fat diet (HFD; cholesterol up to 70%), (B) low-fat diet (LFD; cholesterol up to 10%), or (C) normal rodent diet (C1000 containing less than 5% of total fat), all in form of regular chow pellets and availability of tap water. All rodent diets were purchased from Altromin Company, Lage, Germany.

### 2.2. Forced Treadmill Exercise Training

Animals (series A–C) were randomly assigned to exercise groups and were subjected to running on a two-lane treadmill (Harvard Apparatus, MA, USA) at a speed of 20 m/min for 30 min each day, 5 days/week (total of 6 wks), prior to development of colitis. The treadmill used in our study has an endless conveyor-type belt, driven by a DC servomotor with optical encoder for precise speed control. The animals were separated from each other by opaque partitions. The motor drive electronics permits the user to select any speed from 0 up to 100 m per min. The front of the treadmill is made of dark acrylic so that the animals can run towards the darkened section of the channel.

After 6 weeks of exercise training, the colitis was induced in groups of trained and untrained rats fed different diets by intracolonic administration of 2,4,6-trinitrobenzenesulfonic acid (TNBS, Sigma, Slough, UK) at a dose of 10 mg/kg, dissolved in 50% solution of ethanol as reported in our previous studies [22, 23]. Briefly, the animals were anaesthetized with phenobarbital (60 mg/kg i.p.) and TNBS was administered into the colon in a volume of 0.25 mL per rat at a depth of 8 cm from the rectum with the use of a soft polyethylene catheter. Until the moment of awakening, the rats were positioned in the Trendelenburg position so as to avoid loss of the TNBS solution* via* rectum. Animals in the control group were given 0.9% saline in a volume of 0.25 mL per rat, corresponding to the rats that were administered TNBS. Following the induction of colitis, animals were housed individually, and daily food intake and body weight were monitored. With the induction of colitis, the exercise sessions have stopped. At day 14 from induction of colonic lesions with TNBS, the animals were weighed and anaesthetized to determine CBF using the H_2_-gas clearance technique. The abdominal cavity was opened and, after separation of the colon, the CBF in the areas of the mucosa not affected by inflammatory lesions was measured. CBF was expressed as a percentage of the CBF in the vehicle-control rats without TNBS administration as reported by our group elsewhere [22].

The area of colonic damage were evaluated planimetrically (Morphomat, Carl Zeiss, Berlin, Germany) by two independent researchers. Subsequently, fragments of the colon (2 mm × 10 mm) with colonic lesions were sampled, fixed with formaldehyde, embedded in paraffin, and routinely stained with haematoxylin and eosin (H&E) for histological assessment. The presence and intensity of histological changes were evaluated for the following criteria: presence, area, and depth of ulceration and presence and intensity of inflammatory infiltrations, ulcerations, and fibrosis. The microscopic changes in the colonic mucosa were graded with a compounded histological score including the extent of (1) crypt damage, (2) regeneration, (3) metaplasia/hyperplasia, (4) lamina propria vascular changes, (5) submucosal changes, and (6) presence of inflammatory infiltrates. The sections were graded with a range from 0 to 4 for each of the previous categories and data were analyzed as a normalized compounded score [22, 23].

### 2.3. Determination of Plasma Cytokines and MPO Activity

Immediately after the CBF measurements, a venous blood sample was drawn from the* vena cava* and placed into EDTA-containing vials and used for the determination of plasma IL-1*β*, TNF-*α*, TWEAK, IL-6, leptin, adiponectin, and irisin levels. Briefly, blood was collected and placed into sterile, plastic syringes and kept in ice till centrifugation. The blood samples were centrifuged with the speed of 1000 G for 10 minutes in 15°C temperature and the plasma was stored in −80°C. The plasma TNF-*α*, IL-1*β*, TWEAK, IL-6, leptin, and adiponectin were determined by a solid phase sandwich ELISA (BioSource International Inc., Camarillo, CA, USA) according to the manufacturer's instructions [24]. Plasma irisin protein concentrations were measured using a specific ELISA kit (Phoenix Pharmaceuticals Inc., USA). The fragments of colonic tissue weighing about 200 mg were collected and frozen in −70°C for the determination of MPO activity by ELISA as reported previously [25].

### 2.4. Expression of IL-1*β*, TNF-*α*, and Hif1*α* Transcripts in the Rat Colonic Mucosa Determined by Reverse Transcriptase-Polymerase Chain Reaction (RT-PCR)

The mRNA expression for IL-1*β*, TNF-*α*, and Hif1*α* was determined by RT-PCR in the unchanged colon mucosa of intact rats or those with TNBS colitis fed with different diets. Biopsy samples of colonic mucosa weighing about 200 mg were scraped off from oxyntic mucosa using a slide glass and immediately snap frozen in liquid nitrogen and stored at −80°C until analysis. The total RNA was extracted from the mucosal samples by a guanidium isothiocyanate/phenol chloroform method using a kit from Stratagene (Heidelberg, Germany) according to methods described by Chomczynski and Sacchi [26]. The concentration of RNA in RNase-free Tris-EDTA buffer was measured at absorption of 260 nm wavelengths by spectrophotometry. Five *μ*g of total cellular RNA single-stranded cDNA was generated using StrataScript reverse transcriptase and oligo(dT) primers (Stratagene). The polymerase chain reaction mixture was amplified in a DNA thermal cycler (Perkin-Elmer-Cetus, Norwalk, CT). The nucleotide sequences of the primers used in PCR were as follows: *β*-actin (size of PCR product 764 bp), forward: 5′-TTG TAA CCA ACT GGG ACG ATA TGG-3′, reverse: 5′-GAT CTT GAT CTT CAT GGT GCT AGG-3′; IL-1*β* (size of PCR product 543 bp), forward: 5′-GCT ACC TAT GTC TTG CCC GT-3′, reverse: 5′-GAC CAT TGC TGT TTC CTA GG-3′; TNF-*α* (size of PCR product 295 bp), forward: 5′-TAC TGA ACT TCG GGG TGA TTG GTC C-3′, reverse: 5′-CAG CCT TGT CCC TTG AAG AGA ACC-3′; Hif1*α* (size of PCR product 510 bp), forward: 5′-TCT GGA CTC TCG CCT CTG-3′, reverse 5′-GCT GCC CTT CTG ACT CTG-3′.

PCR products were separated by electrophoresis in 2% agarose gel containing 0.5 *μ*g/mL ethidium bromide and then visualized under UV light as described previously [23, 24]. The signal intensity of expression of mRNAs for IL-1*β*, TNF-*α*, and Hif1*α* was analyzed by densitometry (Gel-Pro Analyzer, Fotodyne Incorporated, Hartland, WI, USA) [24].

### 2.5. Statistical Analysis

Results are expressed as means ± SEM. The data was processed by the statistical analysis software SPSS version 16.0 (SPSS Inc., Chicago, IL). Statistical analysis was done using Student's *t*-test or analysis of variance and two-way ANOVA test with Tukey post hoc test where appropriate. Differences of *P* < 0.05 were considered significant.

## 3. Results

### 3.1. The Effects of Exercise on the Healing Process of Colonic Lesions and CBF in Rats Fed with Different Diet

**Figure 1:**
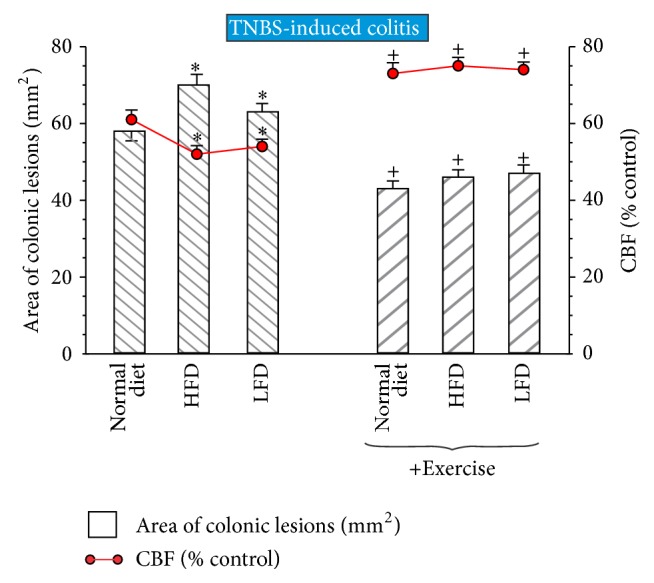
Effect of exercise on the area of colonic lesions and the alterations in CBF in animals fed normal diet, high-fat diet (HFD), and low-fat diet (LFD). Results are mean ± SEM of 8 animals per each group. An asterisk indicates a significant change (*P* < 0.05) as compared to respective values in rats fed normal diet. Cross indicates a significant change (*P* < 0.05) as compared to the respective values obtained in animals fed different diets but not subjected to exercise.

As shown in Figure 1, the intrarectal administration of TNBS in rats induced severe mucosal injury characterized by necrosis of the epithelium and focal ulcerations of the mucosa in rats fed with normal diet. This colonic damage was significantly aggravated in rats with HFD (*P* < 0.05) and also significantly increased in those fed with LFD. The CBF was significantly decreased in HDF and LDF rats (*P* < 0.05) as compared with those fed with normal diet. In rats subjected to exercise prior to administration of TNBS, a significant reduction in the area of colonic lesions (*P* < 0.05) and a significant increase in CBF (*P* < 0.05) were observed as compared to those recorded in sedentary animals with TNBS colitis (Figure 1).

**Figure 2:**
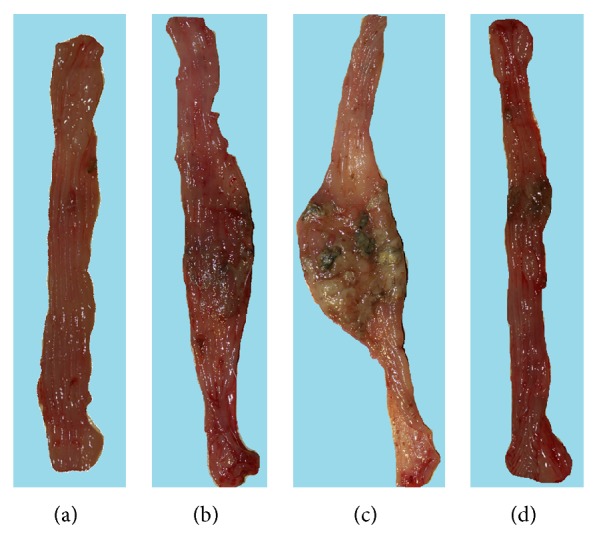
The representative macroscopic appearance of intact colonic mucosa (a) and the colonic mucosa of rat with TNBS colitis fed normal diet (b) or HFD without exercise (c) and HFD with exercise (d) at day 14 after TNBS induction. Note, the normal appearance of colonic mucosa (a) and the severe colonic damage in TNBS rat fed normal diet (b). This damage was exacerbated in untrained rat fed HFD as reflected by necrotic damage occupying larger area of colonic mucosa (c). However, in trained rat fed HFD, the colonic damage was reduced and smaller area of the colonic damage was clearly confirmed by gross inspection (d).

Figures 2(a), 2(b), 2(c), and 2(d) show the macroscopic appearance of the intact colonic mucosa (a) and that with TNBS colitis in rats fed with normal diet (b) or HFD without exercise (c) or HFD with exercise (d). The intact colonic mucosa showed normal macroscopic appearance but, in rats administered with TNBS, the severe colonic damage as manifested by the area of mucosal damage was observed and this damage was markedly exacerbated in rats fed with HFD (Figure 2(b) versus Figure 2(c)). In TNBS rats fed HFD and subjected to exercise, the area of colonic lesions was reduced comparing to that observed in TNBS rats fed HFD without exercise training (Figure 2(d) versus Figure 2(c)).

**Figure 3:**
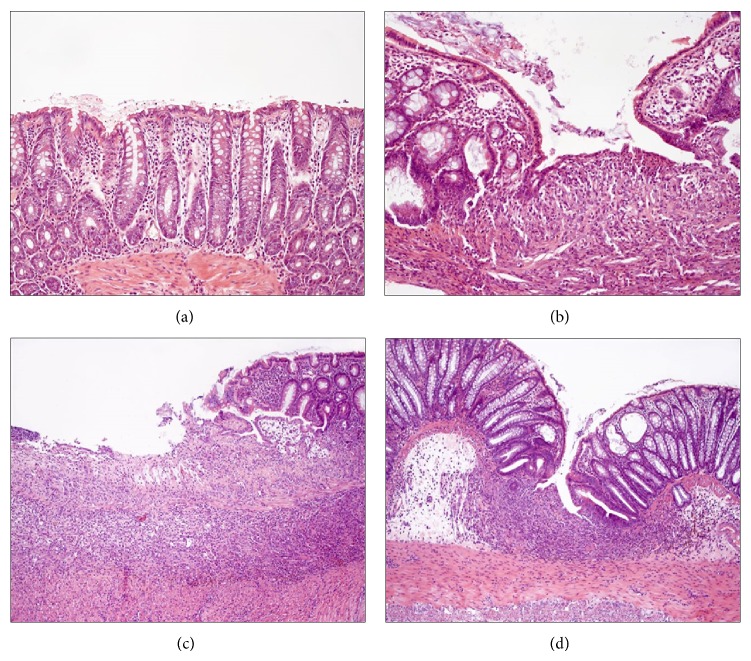
The histological appearance of colonic mucosa of intact rat (a) and the colonic mucosa of rat with TNBS colitis fed normal diet (b) or fed HFD without exercise (c) and HFD with exercise (d) at day 14 after TNBS induction. Note the normal architecture of the colonic crypts in intact rat (a) and the severe damage as manifested by a partial loss of normal architecture and deep ulceration reaching* muscularis mucosa* (b). This histological damage was potentiated in untrained rat fed HFD showing complete loss of architecture accompanied by severe inflammation (c). In contrast, in trained rat fed HFD, both the area of colonic damage and the inflammatory reaction were reduced and more regeneration around the ulceration was evidently seen (d).

Figures 3(a), 3(b), 3(c), and 3(d) show that the representative colonic tissue pathomorphologic changes were evidently more severe in rats with TNBS colitis fed normal diet or those with TNBS colitis fed HFD compared with intact colonic mucosa. The histological appearance of mucosa of rat fed normal diet was illustrated by a desquamation of colonic epithelium and deep ulcerations reaching* muscularis mucosa* followed by neutrophil infiltration. In HFD rats, there was a total disorganization of mucosal structure, deep ulceration with necrosis, and heavy inflammation followed by intense infiltration with neutrophils, fibrosis, and lesser regeneration comparing with rats fed with normal diet. In contrast, the less pronounced histologic changes and neutrophil infiltration were observed in HFD rats subjected to exercise as manifested by the partial restoration of colonic mucosa architecture, a more pronounced regeneration, and the lack of deep mucosal ulcerations indicating more advanced healing of colonic mucosa compared with that in sedentary HFD rats without exercise (Figure 3(d) versus Figure 3(c)).

### 3.2. Effect of Exercise on the Weight of Colon and MPO Activity

**Figure 4:**
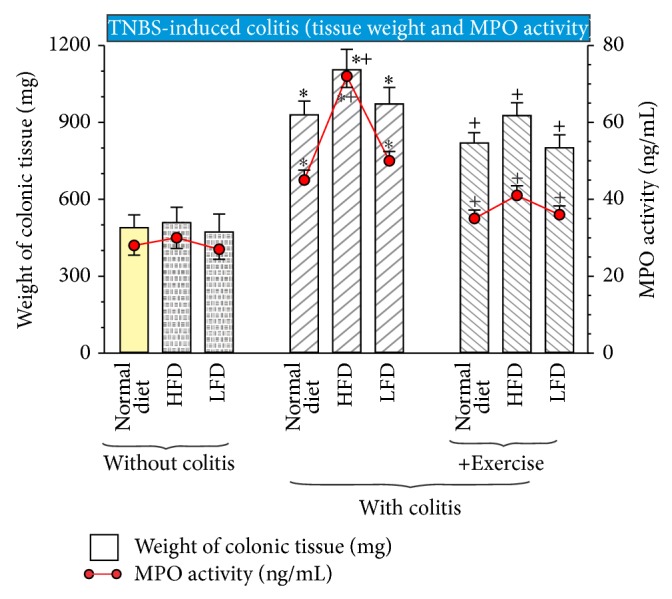
The effect of exercise on the weight of colonic tissue and the colonic MPO levels in rats with or without TNBS colitis fed normal diet, HFD, and LFD. Results are mean ± SEM of seven animals per each group. An asterisk indicates a significant difference (*P* < 0.05) as compared with the control groups of animals (without colitis). An asterisk and cross indicate a significant difference (*P* < 0.05) as compared to the values obtained in rats fed normal diet. Cross indicates significant difference (*P* < 0.05) as compared to the values obtained in TNBS rats fed different diets without exercise.

As shown in Figure 4, the weight of colonic tissue was not significantly affected in rats without colitis fed different diets: either normal diet, HFD, or LFD. However, the weight of examined colonic tissue was significantly increased in rats with TNBS-induced colitis fed with normal diet when compared with that measured in rats without colitis (Figure 4). The weight of colonic tissue in TNBS exercising rats was significantly decreased in comparison with the untrained animals (Figure 4). Comparing with LFD or normal diet, TNBS rats subjected to HFD showed the significant increase in the weight of colonic tissue (*P* < 0.05) (Figure 4). MPO activity, as the marker of neutrophil infiltration of colonic mucosa which corresponded with the intensity of inflammation, was significantly increased in the colonic mucosa of TNBS colitis (*P* < 0.02) as compared with MPO activity in rats without colitis (Figure 4). In HFD rats, a significant elevation of MPO activity over the values measured in rats fed both of the normal diet and LFD (*P* < 0.05) was observed. As shown in Figure 4, in TNBS rats fed HDF and subjected to exercise, a significant fall of MPO activity (*P* < 0.05) in the colonic mucosa comparing with group of sedentary rats fed different diets was recorded.

### 3.3. Effect of Exercise on Plasma Proinflammatory Cytokines IL-1*β*, TWEAK, and TNF-*α* Levels

**Figure 5:**
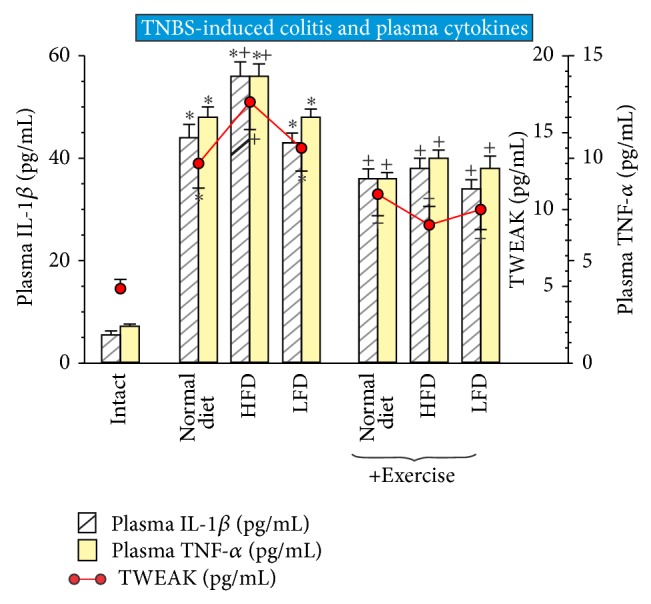
The effect of exercise on the plasma levels of proinflammatory cytokines IL-1β and TNF-α and TWEAK in TNBS rats fed normal diet, HFD, or LFD with or without exercise. Results are mean ± SEM of eight animals per each experimental group. Asterisk indicates a significant change (*P* < 0.05) as compared with respective values in intact rats. Asterisk and cross indicate a significant change (*P* < 0.05) as compared with respective values in animals fed with normal diet. Cross indicates a significant change (*P* < 0.05) as compared with those obtained in untrained animals.

Plasma levels of proinflammatory cytokines IL-1*β*, TWEAK, and TNF-*α* were significantly elevated in animals with TNBS-induced colitis (*P* < 0.02) in comparison with intact rats (Figure 5). In rats fed with HFD, a significant increase in plasma IL-1*β*, TNF-*α*, and TWEAK levels (*P* < 0.05) was observed comparing to normal and LFD group. In exercising TNBS rats, a significant attenuation in plasma levels of proinflammatory cytokines IL-1*β*, TNF-*α*, and TWEAK was observed (*P* < 0.05) as compared to the values of these cytokines in sedentary rats fed with different diets. However, there were no significant differences between HFD, LFD, and normal diet groups in plasma levels of IL-1*β*, TNF-*α*, and TWEAK in TNBS-rats subjected to exercise (Figure 5).

### 3.4. Effect of Exercise on Plasma Adipokine Leptin and Adiponectin Levels

**Figure 6:**
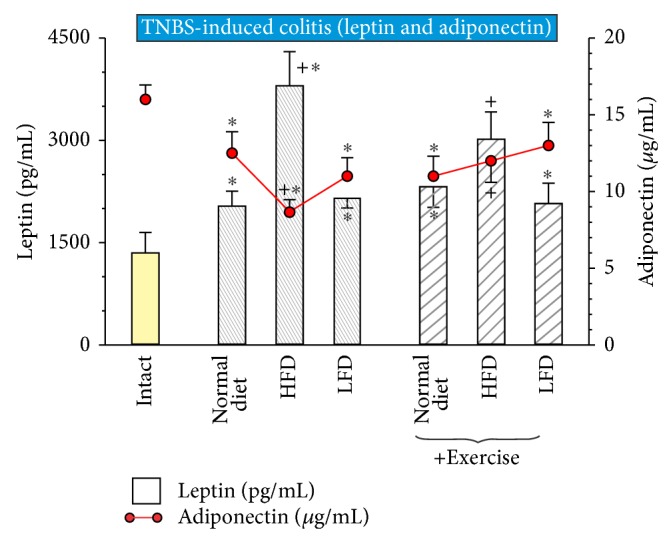
The effect of exercise on the plasma levels of leptin and adiponectin in trained and untrained rats fed normal diet, HFD, or LFD. Results are mean ± S.E.M. of seven animals per each experimental group. Asterisk indicates a significant change (*P* < 0.05) as compared with respective values in intact rats. Asterisk and cross indicate a significant change (*P* < 0.05) as compared with respective values in animals fed with normal diet or LFD. Cross indicates a significant change (*P* < 0.05) as compared with those obtained in untrained animals.

Plasma level of leptin was significantly elevated and the plasma adiponectin level was significantly decreased in animals with TNBS-induced colitis fed normal diet (*P* < 0.05) comparing with those measured in intact animals (Figure 6). HFD significantly increased plasma leptin level and significantly decreased the plasma adiponectin levels (*P* < 0.05) as compared to values obtained in rats fed either normal diet or LDF (Figure 6). Exercise significantly decreased plasma levels of leptin and also significantly increased the plasma adiponectin levels in rats fed HFD (*P* < 0.05) as compared to the values of this adipokine recorded in untrained rats.

### 3.5. Effect of Exercise on Plasma Myokine Irisin and IL-6 Levels in Rats with TNBS Colitis Fed Different Diets with or without Exercise

**Figure 7:**
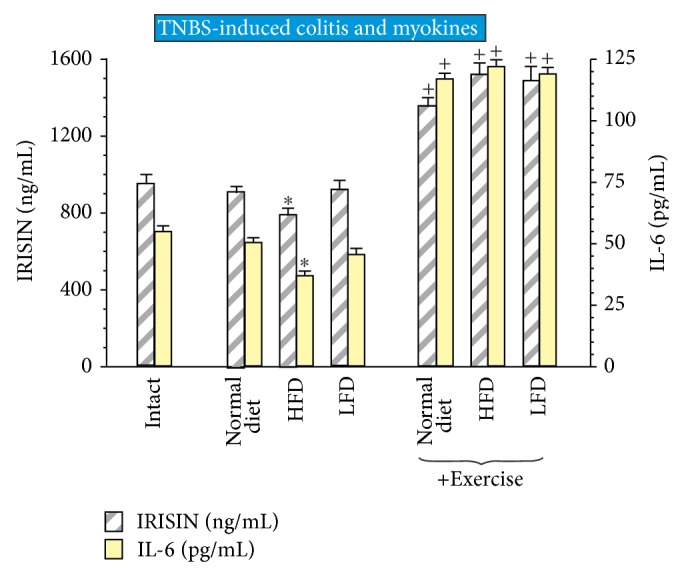
The effect of exercise on the plasma levels of irisin and IL-6 in trained and untrained rats fed normal diet, HFD, or LFD. Results are mean ± SEM of eight animals per each experimental group. Asterisk indicates a significant change (*P* < 0.05) as compared with respective values in intact rats. Cross indicates a significant change (*P* < 0.05) as compared with those obtained in animals not subjected to exercise.

As shown in Figure 7, the plasma levels of irisin and IL-6 were not significantly altered in TNBS rats fed normal diet as compared with intact rats. However, in TNBS colitis rats fed with HFD, the plasma levels of irisin and IL-6 were significantly decreased (*P* < 0.05) as compared with those fed LFD. Exercise significantly increased plasma levels of irisin and IL-6 (*P* < 0.05) in TNBS rats fed normal diet, HFD, and LFD comparing to those recorded in sedentary rats (Figure 7).

### 3.6. Effect of Exercise on the Mucosal Expression of *β*-Actin, IL-1*β*, TNF-*α*, and Hif1*α* in Rats with TNBS Colitis Fed HFD or LFD with or without Exercise

**Figure 8:**
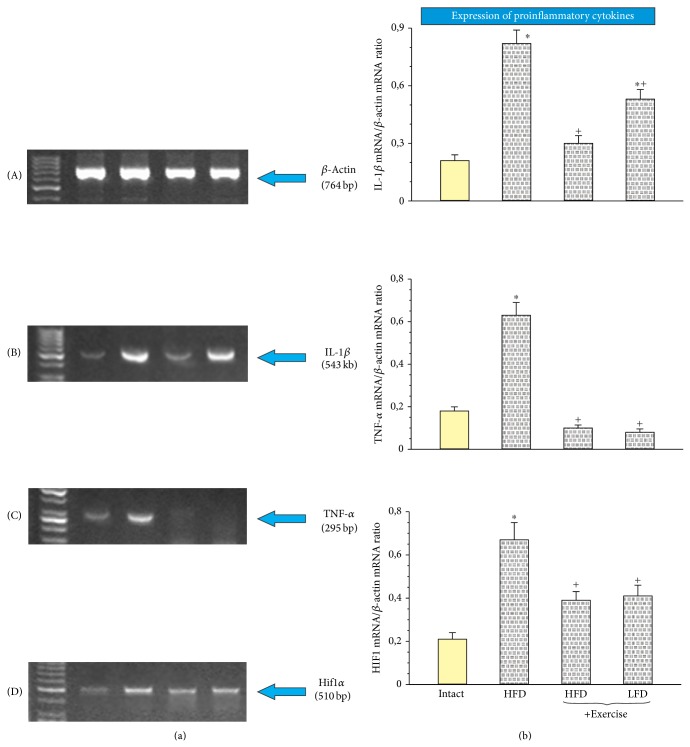
The RT-PCR analysis of mRNA expression for β-actin (A), IL-1β (B), TNF-α (C), and Hif1α (D) (a) in colonic mucosa of intact rats or untrained TNBS rats fed HFD or trained TNBS rats fed HDF or LDF and the semiquantitative ratio of IL-1β-, TNF-α-, and Hif1α mRNAs over β-actin mRNA (b). Results are mean ± SEM of 4 determinations. Asterisk indicates a significant change (*P* < 0.05) as compared with the values obtained in intact colonic mucosa. Cross indicates a significant change (*P* < 0.05) as compared with untrained rats fed with HFD.

Figures 8(A)–8(D) show the effect of HFD alone or HFD and LFD with exercise on the mRNA expression of proinflammatory cytokines IL-1*β* and TNF-*α* and proinflammatory marker Hif1*α* in the colonic mucosa of rats with TNBS-induced colitis fed different diets. The weak signals for expression of IL-1*β*, TNF-*α*, and Hif1*α* mRNAs were recorded in intact mucosa (Figures 8(B), 8(C), and 8(D)). In untrained rats fed HFD, the signal intensity for these factors was significantly enhanced compared to that observed in the intact colonic mucosa (Figure 8(a)). This increase in signal intensity observed in rats fed HFD without exercise was significantly inhibited in trained rats fed HFD. The semiquantitative ratio of IL-1*β*-, TNF-*α*-, and Hif1*α* mRNAs over *β*-actin mRNA confirmed that the IL-1*β*, TNF-*α*, and Hif1*α* mRNAs were upregulated in TNBS rats fed HFD as compared with the expression of mRNA for these factors detected in intact colonic mucosa (Figure 8(b)). The signal intensity of mRNAs expression of IL-1*β*, TNF-*α*, and Hif1*α* in rats fed HFD or LFD was significantly inhibited in those subjected to exercise. The ratio of mRNAs for IL-1*β*, TNF-*α*, and Hif1*α* over *β*-actin mRNA confirmed that the expression of mRNAs for IL-1*β*, TNF-*α*, and Hif1*α* was significantly decreased in TNBS rats subjected to exercise (Figures 8(a) and 8(b)).

## 4. Discussion

The results of our present study indicate that diet-induced obesity delayed the healing of experimental colitis in rats and that the forced moderate treadmill running 6 weeks prior to colitis induction significantly attenuated the severity of colonic damage induced by colonic application of TNBS in these rats. The mechanism of a beneficial effect of exercise observed in our study should be further elucidated but we found that this effect could be attributed to exercise-induced increase in the CBF and the release of myokines that can attenuate the gross, histological, and functional changes in the inflamed colon leading to an improvement in the mucosal healing of colitis. The development of experimental colitis in HFD rats was accompanied by the fall in CBF, the increased expression of proinflammatory cytokines IL-1*β*, TNF-*α*, and TWEAK, and decreased release of protective adipokines (e.g., adiponectin). All of these alterations possibly contributed to the exacerbation of colitis in these rats when compared to those that were fed a normal diet or low fat diet. We provide evidence that exercise applied prior to the development of colitis, especially in rats with diet-induced obesity as reflected by an increase in weight of colonic tissue and increased MPO activity, resulted in an enhancement in colonic microcirculation as a conglomeration of an increase in CBF, lower colonic tissue weight, an increase of MPO content, and the increased plasma levels of protective myokines, irisin, and IL-6. Moreover, exercise attenuated the expression of proinflammatory cytokines IL-1*β* and TNF-*α* and their plasma levels, as well as the expression of inflammatory state and cytokines of the colonic mucosa as well as Hif1*α*, in rats subjected to HFD, which suggested that moderate exercise selected in our study exerts a beneficial influence on the healing of colonic mucosa in this rodent model of colitis.

It is now generally accepted that obesity represents a low-grade chronic inflammatory state, characterized by abnormal profile of cytokine secretion, increased synthesis of acute-phase reactants, such as C-reactive protein (CRP), and the activation of proinflammatory signaling pathways [27]. Adipocytes are now recognized as new members of the immune system, producing several cytokines such as IL-6, TNF-*α*, and chemokines, in addition to adipokines (leptin, adiponectin, and resistin) [28, 29]. Abdominal adiposity and especially mWAT has been implicated in a wide range of gastrointestinal disorders from fatty liver and GI cancers to acute pancreatitis and Crohn's disease (CD) [30]. Furthermore, the macrophages infiltrate adipose tissue during obesity, thus contributing to production and release of additional inflammatory mediators [31], and the adipose tissue depots can be altered due to inflammatory pathologies such as CD [11, 14, 28]. Interestingly, CD has not necessarily been associated with obesity and is characterized by hypertrophy of mesenteric adipose tissue [14]. Several mechanisms could be responsible for a link of general or local obesity with CD. In genetically obese mice, the increased intestinal permeability has been described and considered one of the major pathogenic mechanisms linked with CD and UC [19].

In the normal intestinal mucosa, a continuous low-grade, nonpathogenic inflammation is observed, and this is triggered by a perpetual exposure of intestinal mucosa to a marked antigenic load from dietary and microbial antigens and several ligands of toll-like receptors (TLRs) [32]. Despite the* lamina propria* infiltration with activated immune cells, the IBD might be developed due to disruption or weakening of the mucosal barrier and recognition of the normal microbiota as pathogens [32]. Both CD and UC patients exhibited the activation of innate (macrophage, neutrophils) and acquired (T and B cell) immune responses and loss of tolerance to enteric commensal bacteria [33]. Fat wrapping and mesenteric adipose tissue hypertrophy are consistent features recognized on surgical specimens in patients with CD [9] and recent research suggests that the hypertrophied fat contributes actively to disease severity and may influence onset of complications [10–16]. It is likely that the mesenteric fat in CD is exposed to gut microbial antigens. Adipocytes express toll-like receptors and CD14, and both were shown to interact with these microbial antigens activating NF-*κ*B pathways [34]. About 95% of the total viable bacteria cultured from mesenteric tissues are physiologically located in adipocytes, and only 5% are translocated in mesenteric lymph nodes, indicating that adipocytes might be a main reservoir of bacteria in the mesentery. Intriguingly, obesity is associated with reduced microbial diversity in a similar pattern to that seen in CD [35, 36]. All these observations fuelled speculation about the potential roles of mesenteric mWAT in the development of CD by reacting to the microbial environment and by initiating and/or promoting local inflammatory reactions by autocrine and/or paracrine modulation of adipocytes [10, 13].

In our study, the impaired healing of TNBS colitis accompanied by the reduction in the colonic blood flow was observed in rats fed HFD compared with rats fed a normal diet or LFD. These changes were accompanied by increase in plasma levels of proinflammatory mediators such as IL-1*β*, TNF-*α*, TWEAK, and leptin and the reduction in plasma adiponectin levels. Particularly interesting to the study, is that the plasma TWEAK level was increased in rats with TNBS-induced colitis fed normal diet and this increase was potently elevated in rats fed HFD. This observation is consistent with the involvement of TWEAK cytokine in the inflammatory processes [37]. TWEAK actions are mediated by its binding to fibroblast growth factor-inducible 14 (Fn14), a highly inducible cell surface receptor that has been linked to several intracellular proinflammatory signaling pathways, including the NF-*κ*B pathway [17]. An increase in TWEAK and Fn14 gene expression in adipose tissue of severely obese patients was reported and inflammatory stimuli* in vitro* differentially increased the expression of TWEAK in macrophages and Fn14 in adipocytes [38]. Expression of Fn14 is upregulated in CD patients [37, 39] and the TWEAK/Fn14 pathway plays a pathological role in mouse models of CD by inducing inflammatory responses and regulating intestinal epithelial cell turnover [39–42].

Furthermore, we found that leptin, the product of adipose tissue, was increased in rats with colitis fed normal diet and this effect was markedly enhanced in rats fed HFD. This remains in agreement with previous observation that intestinal leptin, a cytokine produced by adipocytes, is increased in CD and can upregulate NF*κ*B expression in colonic epithelial cells leading to development of inflammation [12, 43, 44]. Leptin is considered to be a proinflammatory cytokine and directly regulates production of several cytokines, particularly those produced by T cells [12]. An overexpression of leptin mRNA in mWAT was reported in IBD patients, indicating that leptin might participate in the inflammatory process by enhancing mesenteric expression of TNF-*α* [45]. Also, in rat model of experimental colitis, an elevated plasma leptin levels were observed which correlated with the degree of inflammation [46]. We also observed that LFD feeding increased the severity of experimental colitis although not to the same extent as observed in case of HFD.

It is of interest that plasma level of adiponectin was significantly decreased in HFD animals than in those fed normal diet or LFD. Adiponectin has a structure similar to TNF-*α* but antagonizes its effects by reducing secretion and attenuating the biological actions by competing for the receptor [47]. Conflicting data have been described for circulating levels of adiponectin in patients with IBD [48–50]. However, recent observations of lower levels of serum and mesenteric adiponectin in active CD patients but not those in remission support the notion of a defective regulation of anti-inflammatory pathways in CD pathogenesis [48]. The altered balance between proinflammatory and anti-inflammatory factors (increase in secretion of TNF-*α*, leptin, and release of chemoattractant protein-1 (MCP-1)) and decreased production of anti-inflammatory factors (e.g., adiponectin) could contribute to macrophage accumulation in adipocytes, as well as to an inflammatory transformation of the visceral adipose tissue, leading to the appearance of creeping fat. Moreover, the depletion of muscle mass and impaired muscle function are important features of IBD [51]. The IBD-related decreased muscle mass has been attributed to a variety of mechanisms including decreased nutrient intake, their malabsorption increased metabolic rate, and the inhibitory effects of inflammation on the growth hormone (GH)/insulin-like growth factor- (IGF-) I axis [52]. TWEAK has recently been shown to mediate the skeletal muscle atrophy in a variety of clinical settings [53]. Gruber et al. [8] observed in CD-relevant mouse model that HFD feeding, independently of obesity, accelerated disease onset of intestinal inflammation through mechanisms involving the increased intestinal permeability and altered luminal factors, leading to enhanced dendritic cell recruitment and promoted Th17 immune responses.

The potential benefits of exercise and physical activity in IBD patients recently raised major interest [54, 55]. Evidence suggests that the protective effect of exercise may to some extent be ascribed to its anti-inflammatory effects and/or specific effects on visceral fat mediated, in part,* via* muscle-derived peptides, so-called “myokines” [19, 56]. Presently identified myokines which exert endocrine, paracrine, or autocrine effects are LIF, IL-6, IL-7, BDNF, IGF-1, FGF-2, FSTL-1, and irisin [57]. The dysfunction of several organs and tissues of the body as well as an increased risk of chronic inflammatory diseases has been linked with lack of the endocrine and paracrine functions of the muscle that are not activated through contractions [57]. Myokines may balance and counteract the effects of adipokines such as leptin taking part in crosstalk between skeletal muscle and adipose tissue [57, 58].

Here we have shown for the first time that the plasma level of irisin was diminished in rats with TNBS-induced colitis fed HFD but not in those fed normal diet or LFD. Exercise training leads to marked increase in plasma irisin levels confirming that irisin identified as a putative myokine that is induced by exercising muscles could be involved in the mechanism of exercise-induced improvement of mucosal healing of colitis observed in our study. Interestingly, the circulating levels of irisin were also negatively associated with obesity and insulin resistance [59]. It was suggested that irisin could be therapeutic for human metabolic disease, obesity, and other disorders in which adipose tissue plays pathogenic role, and the exercise was found to exert beneficial influence [60]. We have also observed significant increases in circulating myokine IL-6 after exercise training. While typically regarded as a proinflammatory cytokine, IL-6 appears to mediate also metabolic effects associated with exercise. Until recently, it was accepted that the rise in plasma IL-6 level observed during exercise was a consequence of immune response to local damage. Today, it is known that muscle is unique in its ability to produce IL-6 during contraction in completely TGF-independent mode, which suggests a major role for this cytokine in a mechanism of regulation of metabolism rather than for this cytokine acting as an inflammatory mediator.

Our results are in variance with those observed in recent studies on the effects of exercise training in a mouse model of colitis [61]. The moderate forced treadmill running exacerbated dextran sodium sulfate- (DSS-) induced experimental colitis while the voluntary wheel running alleviated colitis symptoms and reduced inflammatory gene expression in these mice [61]. These contradictory effects of voluntary and forced exercise could be explained by effect of stress resulting from the extensive forced exercise in comparison with voluntary exercise. The differences between the results of our study and those by Cook et al. [61] could be explained by differences in experimental model of colitis and the bout of exercise used in both studies in different animal species. In the case of rats subjected to exercise, the applied intensity of forced exercise could be relatively smaller than for mice. Intensive exercise could lead to transient systemic inflammation and increased level of proinflammatory cytokines. It is well known that intensive exercise in humans could cause nausea, diarrhea, and gastrointestinal bleeding. For instance, in marathon runners, the disorder called “runner's ischemic colitis” associated with bloody diarrhea, fatigue, and fever has been described [62]. Therefore, the effects of physical exercise on various immune parameters during the course of IBD may depend on type of exercise and its intensity and duration. Systematic, moderate exercise may be beneficial for IBD patients with respect to exercise-induced anti-inflammatory and anabolic mechanisms. In contrast, the acute intensive exercise may result in a release of proinflammatory cytokines predominantly in obese individuals leading to exacerbation of the inflammatory response and worsening of the pathology of CD [54].

## 5. Conclusions

Diet-induced obesity delays the healing of experimental colitis* via* decrease in CBF and the increase in MPO activity and the expression and release of proinflammatory mediators. Exercise diminished the severity of colonic damage mediated, at least in part, by an improvement of colonic microcirculation, the release of myokines such as protective irisin, and the restoration of adipokine adiponectin. We propose that the regular exercise could exert a beneficial effect in human IBD as documented by our translational research and accumulated evidence in experimental rodent colitis. This beneficial effect of exercise may be mediated by its long-term effects on abdominal adiposity and the anti-inflammatory environment that is created by each acute bout of exercise.

## References

[1] Baumgart D. C., Sandborn W. J. (2007). Inflammatory bowel disease: clinical aspects and established and evolving therapies. *The Lancet*.

[2] Sartor R. B. (2006). Mechanisms of disease: pathogenesis of Crohn's disease and ulcerative colitis. *Nature Clinical Practice Gastroenterology and Hepatology*.

[3] Strober W., Fuss I. J. (2011). Proinflammatory cytokines in the pathogenesis of inflammatory bowel diseases. *Gastroenterology*.

[4] Strober W., Zhang F., Kitani A., Fuss I., Fichtner-Feigl S. (2010). Proinflammatory cytokines underlying the inflammation of Crohn's disease. *Current Opinion in Gastroenterology*.

[5] Cosnes J., Gowerrousseau C., Seksik P., Cortot A. (2011). Epidemiology and natural history of inflammatory bowel diseases. *Gastroenterology*.

[6] Bertin B., Desreumaux P., Dubuquoy L. (2010). Obesity, visceral fat and Crohn's disease. *Current Opinion in Clinical Nutrition and Metabolic Care*.

[7] Suibhne T. N., Raftery T. C., McMahon O., Walsh C., O'Morain C., O'Sullivan M. (2013). High prevalence of overweight and obesity in adults with Crohn's disease: associations with disease and lifestyle factors. *Journal of Crohn's and Colitis*.

[8] Gruber L., Kisling S., Lichti P. (2013). High fat diet accelerates pathogenesis of murine Crohn's disease-like ileitis independently of obesity. *PLoS ONE*.

[9] Sheehan A. L., Warren B. F., Gear M. W. L., Shepherd N. A. (1992). Fat-wrapping in Crohn's disease: pathological basis and relevance to surgical practice. *British Journal of Surgery*.

[10] Peyrin-Biroulet L., Gonzalez F., Dubuquoy L. (2012). Mesenteric fat as a source of C reactive protein and as a target for bacterial translocation in Crohn's disease. *Gut*.

[11] Peyrin-Biroulet L., Chamaillard M., Gonzalez F. (2007). Mesenteric fat in Crohn's disease: a pathogenetic hallmark or an innocent bystander?. *Gut*.

[12] Kaser A., Tilg H. (2012). ‘Metabolic aspects’ in inflammatory bowel diseases. *Current Drug Delivery*.

[13] Batra A., Heimesaat M. M., Bereswill S. (2012). Mesenteric fat—control site for bacterial translocation in colitis?. *Mucosal Immunology*.

[14] Fink C., Karagiannides I., Bakirtzi K., Pothoulakis C. (2012). Adipose tissue and inflammatory bowel disease pathogenesis. *Inflammatory Bowel Diseases*.

[15] Drouet M., Dubuquoy L., Desreumaux P., Bertin B. (2012). Visceral fat and gut inflammation. *Nutrition*.

[16] Acedo S. C., Gotardo É. M. F., Lacerda J. M., de Oliveira C. C., de Oliveira Carvalho P., Gambero A. (2011). Perinodal adipose tissue and mesenteric lymph node activation during reactivated TNBS-colitis in rats. *Digestive Diseases and Sciences*.

[17] Kredel L. I., Batra A., Stroh T. (2013). Adipokines from local fat cells shape the macrophage compartment of the creeping fat in Crohn's disease. *Gut*.

[18] Wilund K. R. (2007). Is the anti-inflammatory effect of regular exercise responsible for reduced cardiovascular disease?. *Clinical Science*.

[19] Pedersen B. K. (2011). Exercise-induced myokines and their role in chronic diseases. *Brain, Behavior, and Immunity*.

[20] Te Velde A. A., Verstege M. I., Hommes D. W. (2006). Critical appraisal of the current practice in murine TNBS-induced colitis. *Inflammatory Bowel Diseases*.

[21] Puleo F., Meirelles K., Navaratnarajah M. (2010). Skeletal muscle catabolism in trinitrobenzene sulfonic acid-induced murine colitis. *Metabolism: Clinical and Experimental*.

[22] Zwolinska-Wcislo M., Brzozowski T., Ptak-Belowska A. (2011). Nitric oxide-releasing aspirin but not conventional aspirin improves healing of experimental colitis. *World Journal of Gastroenterology*.

[23] Zwolinska-Wcislo M., Krzysiek-Maczka G., Ptak-Belowska A. (2011). Antibiotic treatment with ampicillin accelerates the healing of colonic damage impaired by aspirin and coxib in the experimental colitis. Importance of intestinal bacteria, colonic microcirculation and proinflammatory cytokines. *Journal of Physiology and Pharmacology*.

[24] Magierowski M., Jasnos K., Pawlik M. (2013). Role of angiotensin-(1–7) in gastroprotection against stress-induced ulcerogenesis. The involvement of mas receptor, nitric oxide, prostaglandins, and sensory neuropeptides. *The Journal of Pharmacology and Experimental Therapeutics*.

[25] Zwolinska-Wcislo M., Brzozowski T., Budak A. (2009). Effect of Candida colonization on human ulcerative colitis and the healing of inflammatory changes of the colon in the experimental model of Colitis ulcerosa. *Journal of Physiology and Pharmacology*.

[26] Chomczynski P., Sacchi N. (1987). Single-step method of RNA isolation by acid guanidinium thiocyanate-phenol-chloroform extraction. *Analytical Biochemistry*.

[27] Yudkin J. S., Stehouwer C. D. A., Emeis J. J., Coppack S. W. (1999). C-reactive protein in healthy subjects: associations with obesity, insulin resistance, and endothelial dysfunction: a potential role for cytokines originating from adipose tissue?. *Arteriosclerosis, Thrombosis, and Vascular Biology*.

[28] Karagiannides I., Pothoulakis C. (2007). Obesity, innate immunity and gut inflammation. *Current Opinion in Gastroenterology*.

[29] Lago F., Dieguez C., Gómez-Reino J., Gualillo O. (2007). Adipokines as emerging mediators of immune response and inflammation. *Nature Clinical Practice Rheumatology*.

[30] Kershaw E. E., Flier J. S. (2004). Adipose tissue as an endocrine organ. *The Journal of Clinical Endocrinology and Metabolism*.

[31] Clement K., Langin D. (2007). Regulation of inflammation-related genes in human adipose tissue. *Journal of Internal Medicine*.

[32] MacDonald T. T., Monteleone I., Fantini M. C., Monteleone G. (2011). Regulation of homeostasis and inflammation in the intestine. *Gastroenterology*.

[33] Mow W. S., Vasiliauskas E. A., Lin Y.-C. (2004). Association of antibody responses to microbial antigens and complications of small bowel Crohn's disease. *Gastroenterology*.

[34] Lin Y., Lee H., Berg A. H., Lisanti M. P., Shapiro L., Scherer P. E. (2000). The lipopolysaccharide-activated Toll-like receptor (TLR)-4 induces synthesis of the closely related receptor TLR-2 in adipocytes. *The Journal of Biological Chemistry*.

[35] Qin J., Li R., Raes J. (2010). A human gut microbial gene catalogue established by metagenomic sequencing. *Nature*.

[36] Turnbaugh P. J., Hamady M., Yatsunenko T. (2009). A core gut microbiome in obese and lean twins. *Nature*.

[37] Burkly L. C., Michaelson J. S., Zheng T. S. (2011). TWEAK/Fn14 pathway: an immunological switch for shaping tissue responses. *Immunological Reviews*.

[38] Vendrell J., Maymó-Masip E., Tinahones F. (2010). Tumor necrosis-like weak inducer of apoptosis as a proinflammatory cytokine in human adipocyte cells: up-regulation in severe obesity is mediated by inflammation but not hypoxia. *Journal of Clinical Endocrinology and Metabolism*.

[39] Kawashima R., Kawamura Y. I., Oshio T. (2011). Interleukin-13 damages intestinal mucosa via TWEAK and Fn14 in mice—a pathway associated with ulcerative colitis. *Gastroenterology*.

[40] Son A., Oshio T., Kawamura Y. I. (2013). TWEAK/Fn14 pathway promotes a T helper 2-type chronic colitis with fibrosis in mice. *Mucosal Immunology*.

[41] Dohi T., Burkly L. C. (2012). The TWEAK/Fn14 pathway as an aggravating and perpetuating factor in inflammatory diseases; focus on inflammatory bowel diseases. *Journal of Leukocyte Biology*.

[42] Dohi T., Borodovsky A., Wu P. (2009). TWEAK/Fn14 pathway: a nonredundant role in intestinal damage in mice through a TWEAK/intestinal epithelial cell axis. *Gastroenterology*.

[43] Olivier I., Théodorou V., Valet P. (2011). Is Crohn’s creeping fat an adipose tissue?. *Inflammatory Bowel Diseases*.

[44] Ponemone V., Keshavarzian A., Brand M. I. (2010). Apoptosis and inflammation: role of adipokines in inflammatory bowel disease. *Clinical and Translational Gastroenterology*.

[45] Barbier M., Vidal H., Desreumaux P. (2003). Overexpression of leptin mRNA in mesenteric adipose tissue in inflammatory bowel diseases. *Gastroenterologie Clinique et Biologique*.

[46] Barbier M., Cherbut C., Aubé A. C., Blottière H. M., Galmiche J. P. (1998). Elevated plasma leptin concentrations in early stages of experimental intestinal inflammation in rats. *Gut*.

[47] Chandran M., Phillips S. A., Ciaraldi T., Henry R. R. (2003). Adiponectin: more than just another fat cell hormone?. *Diabetes Care*.

[48] Rodrigues V. S., Milanski M., Fagundes J. J. (2012). Serum levels and mesenteric fat tissue expression of adiponectin and leptin in patients with Crohn's disease. *Clinical and Experimental Immunology*.

[49] Weigert J., Obermeier F., Neumeier M. (2010). Circulating levels of chemerin and adiponectin are higher in ulcerative colitis and chemerin is elevated in Crohn's disease. *Inflammatory Bowel Diseases*.

[50] Valentini L., Wirth E. K., Schweizer U. (2009). Circulating adipokines and the protective effects of hyperinsulinemia in inflammatory bowel disease. *Nutrition*.

[51] Shanahan F. (2002). Crohn's disease. *The Lancet*.

[52] Shamir R., Phillip M., Levine A. (2007). Growth retardation in pediatric Crohn's disease: pathogenesis and interventions. *Inflammatory Bowel Diseases*.

[53] Bhatnagar S., Mittal A., Gupta S. K., Kumar A. (2012). TWEAK causes myotube atrophy through coordinated activation of ubiquitin-proteasome system, autophagy, and caspases. *Journal of Cellular Physiology*.

[54] Narula N., Fedorak R. N. (2008). Exercise and inflammatory bowel disease. *Canadian Journal of Gastroenterology*.

[55] Pérez C. A. (2009). Prescription of physical exercise in Crohn's disease. *Journal of Crohn's and Colitis*.

[56] Pedersen B. K. (2011). Muscles and their myokines. *Journal of Experimental Biology*.

[57] Pedersen B. K., Febbraio M. A. (2012). Muscles, exercise and obesity: skeletal muscle as a secretory organ. *Nature Reviews Endocrinology*.

[58] Bilski J., Mazur-Bialy A. I., Wierdak M., Brzozowski T. (2013). The impact of physical activity and nutrition on inflammatory bowel disease: the potential role of cross talk between adipose tissue and skeletal muscle. *Journal of Physiology and Pharmacology*.

[59] Moreno-Navarrete J. M., Ortega F., Serrano M. (2013). Irisin is expressed and produced by human muscle and adipose tissue in association with obesity and insulin resistance. *Journal of Clinical Endocrinology and Metabolism*.

[60] Boström P., Wu J., Jedrychowski M. P. (2012). A PGC1-*α*-dependent myokine that drives brown-fat-like development of white fat and thermogenesis. *Nature*.

[61] Cook M. D., Martin S. A., Williams C. (2013). Forced treadmill exercise training exacerbates inflammation and causes mortality while voluntary wheel training is protective in a mouse model of colitis. *Brain, Behavior, and Immunity*.

[62] Ho G. W. K. (2009). Lower gastrointestinal distress in endurance athletes. *Current Sports Medicine Reports*.

